# Functional Role of NBS1 in Radiation Damage Response and Translesion DNA Synthesis

**DOI:** 10.3390/biom5031990

**Published:** 2015-08-20

**Authors:** Yuichiro Saito, Kenshi Komatsu

**Affiliations:** Genome Repair Dynamics, Radiation Biology Center, Kyoto University, Yoshida Konoe, Sakyo-ku, Kyoto 606-8501, Japan; E-Mail: komatsu@house.rbc.kyoto-u.ac.jp

**Keywords:** NBS1, DNA repair, homologous recombination, chromatin remodeling, translesion DNA synthesis

## Abstract

Nijmegen breakage syndrome (NBS) is a recessive genetic disorder characterized by increased sensitivity to ionizing radiation (IR) and a high frequency of malignancies. NBS1, a product of the mutated gene in NBS, contains several protein interaction domains in the N-terminus and C-terminus. The C-terminus of NBS1 is essential for interactions with MRE11, a homologous recombination repair nuclease, and ATM, a key player in signal transduction after the generation of DNA double-strand breaks (DSBs), which is induced by IR. Moreover, NBS1 regulates chromatin remodeling during DSB repair by histone H2B ubiquitination through binding to RNF20 at the C-terminus. Thus, NBS1 is considered as the first protein to be recruited to DSB sites, wherein it acts as a sensor or mediator of DSB damage responses. In addition to DSB response, we showed that NBS1 initiates Polη-dependent translesion DNA synthesis by recruiting RAD18 through its binding at the NBS1 C-terminus after UV exposure, and it also functions after the generation of interstrand crosslink DNA damage. Thus, NBS1 has multifunctional roles in response to DNA damage from a variety of genotoxic agents, including IR.

## 1. Introduction

Nijmegen breakage syndrome (NBS) is a recessive genetic disorder characterized by immunodeficiency, microcephaly, growth retardation, and a high frequency of malignancies [1]. Cells derived from patients with NBS exhibit high sensitivity to DNA-damaging agents, including ionizing radiation (IR), chromosome instability, and abnormal cell cycle checkpoints [2]. We had successfully mapped the candidate region of the underlying gene into 8q21–24 [3], and from this region, we and other researchers identified the NBS1 gene that is comprised of 16 exons spanning a genomic DNA of 50 kb [4,5,6]. The 657del5 of NBS1 is found in more than 90% of the patients with NBS that slightly expresses a C-terminal fragment (p70). Although more than 10 mutations have been reported in the patients with NBS, the majority of them expressed either p70 or p50, a truncated protein of the C-terminus, with some residual functions (bi-allelic hypomorphic mutation) [7]. Similarly, viable cells from a NBS1-deficient mouse expressed a truncated protein of the C-terminus, which corresponds to human p70 or p50 [8].

Double-strand breaks (DSBs) are considered to be among the most lethal forms of DNA damage because one unrepaired DSB is sufficient to elicit permanent growth arrest or cell death [9]. The majority of DSBs are rejoined by either of the following two DSB repair pathways: the non-homologous end-joining (NHEJ) pathway and the homologous recombination (HR) repair pathway [10,11]. To optimize DSB repair, an appropriate spatiotemporal regulation is provided through the machineries of cell cycle checkpoints [12,13] and chromatin remodeling which are coordinated by several players of the DSB response, termed as sensor/mediator, transducer, and effector. Thus, a fine-tuning cascade is initiated by the formation of a large protein complex, the so-called radiation-induced nuclear foci, at DSB sites [14,15,16,17]. NBS1, as a complex with MRE11 and RAD50 (MRN complex), is one of the first proteins to form radiation-induced nuclear foci. NBS1 acts on this foci as a damage sensor/mediator that recruits the key transducer ATM kinase to DSB sites, although an initial activation of ATM possibly occurs outside DSB sites [18]. The large complex formation of repair factors, such as the mediator of DNA damage checkpoint 1 (MDC1), TopBP1, WRN, and phosphorylated histone H2AX (γH2AX), leads to further activation of ATM kinase in a positive feedback loop so that DSB signals are amplified and transduced to numerous downstream effectors, including p53 for cell cycle checkpoints, CHD3.1 for chromatin remodeling, and Rad51 for DSB repair machinery [12].

NBS1 is implicated in the maintenance of genome integrity after many insults to prevent malignancy, particularly lymphoma, because endogenous DSB is generated during V(D)J recombination in lymphoblastoid cells. However, it has been reported that heterozygous carriers with an NBS1 mutation develop other types of malignancies such as melanoma. In the first report on patients, NBS was characterized as a syndrome with high sensitivity to sunlight. Similarly, another patient with NBS was misdiagnosed as having Fanconi anemia (FA) because the cells from this patient were sensitive to DNA interstrand crosslink (ICL) agents such as mitomycin-C; this feature is a hallmark of FA. This evidence suggests that NBS1 has multifunctional roles in response to DNA damage from a variety of genotoxic agents. Here, we summarize the functional roles of NBS1 in response to ultraviolet (UV) and ICLs in addition to IR.

## 2. Functional Domains of NBS1

**Figure 1:**
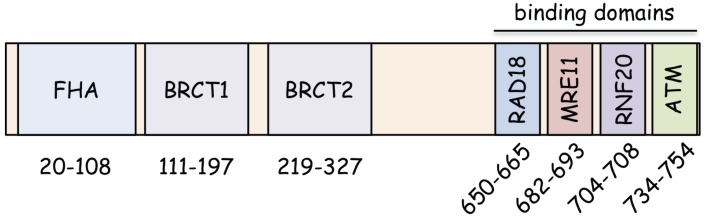
The structure of human NBS1. NBS1 contains several interaction domains in the N- and C-termini.

NBS1 represents several functional domains in the N-terminus and C-terminus (Figure 1), some of which were discovered to have a weak homology to the Xrs2 protein, a budding yeast homolog of NBS1. A fork head-associated (FHA) domain (20–108 residues) and two BRCA1 C-terminus (BRCT) domains (BRCT1, 111–197 residues; BRCT2, 219–327 residues) are located at the N-terminus of NBS1, although Xrs2 lacks the BRCT2 domain. NBS1 binds to CtIP (yeast Sae2 or Ctp1) through the FHA domain for subsequent DNA end resection during HR repair when CtIP is phosphorylated on threonine. Similarly, both FHA and BRCT1/2 domains interact with several phosphoserines in the tandem Ser-Asp-Thr (SDT) motifs of MDC1 for the formation of radiation-induced nuclear foci at DSB sites [16]. This structural mechanism of the manner in which the N-terminus in NBS1 binds to both CtIP and MDC1 through shared phosphopeptide motifs is clearly shown using high-resolution X-ray crystallography analysis [19]. Interestingly, malignances observed in patients with NBS can be attributed to the lack of FHA and BRCT1/2 domains. Indeed, patients with a hypomorphic mutation lacking FHA and BRCT1/2 domains (p70) develop malignances at a median age of 9.5 years, whereas patients expressing p80, which contains FHA and BRCT1/2 domains, never develop any malignancy [20].

NBS1 binds to MRE11 at the C-terminus, thereby forming the MRE11/RAD50/NBS1 (MRN) complex, which plays a role in HR repair [21,22]. This is comparable to the scMre11/scRAD50/Xrs2 (MRX) complex in yeast, which has critical roles in DSB repair and meiotic recombination [23,24,25]. Although sequence homology with yeast Xrs2 was not found, several regions of protein interaction were identified at the NBS1 C-terminus. Falck *et al.* reported an ATM-binding domain at the extreme C-terminus of NBS1, in which NBS1 directly interacts with ATM to recruit it to DSB sites [26]. Our yeast two-hybrid assay identified another interaction region (704–708 residues) adjacent to the MRE11-binding domain, wherein NBS1 binds to E3 ubiquitin ligase RNF20 (yeast homolog of Bre1) for histone H2B ubiquitination and regulates chromatin remodeling [27,28] (see Section 5). In addition to these domains, we also found a novel binding domain at the C-terminus of NBS1 (650–665 residues), wherein NBS1 interacts with the E3 ubiquitin ligase RAD18 to initiate translesion DNA synthesis (TLS) by proliferating cell nuclear antigen (PCNA) ubiquitination after UV exposure [8] (see Section 6). Thus, interaction domains at the C-terminus are well conserved among vertebrates, but they, with the exception of the MRE11-binding domain, are not conserved in yeast. The absence of the C-terminus causes cell death, whereas p70 containing the C-terminus with some residual function ensures survival [29].

## 3. A Role of NBS1 in HR Repair

It has been well established that the yeast MRX complex plays a role in DSB repair via both HR and NHEJ [30]. To investigate the roles of NBS1 complex in vertebrate DSB repair, we developed an *Nbs1*-deficient DT40, a chicken B-cell line, because any of the NBS1-, MRE11-, or RAD50-knockout mice are lethal in the embryonic stages [31,32]. As expected, the *Nbs1*-deficient DT40 cells exhibited high sensitivity to IR and a marked reduction of sister chromatid exchanges after treatment with mitomycin-C, suggesting an involvement of NBS1 in HR repair [21]. This defect in HR repair was confirmed by the HR reporter gene assay using SCneo, which showed considerable reduction (approximately 200-fold) in the frequency of HR repair in the *Nbs1*-deficient DT40 cells as compared with wild-type cells [33]. These findings are consistent with the observations that meiotic recombination and HR repair are defective in *h2ax* knockout mice, *i.e.*, NBS1 was unable to accumulate at DSB sites [34,35]. HR repair is initiated with the processing of DNA ends to produce 3' single-stranded tails, followed by strand invasion and pairing with homologous DNA sequences of sister chromatids during the S and G2 phases [36,37,38,39]. The fission yeast spNbs1 promotes DNA end resection by interaction with Mre11 and Ctp1, the yeast homologs of CtIP [19,40]. Upon measurement of replication protein A (RPA) accumulation, the mutation of human NBS1 at the CDK-mediated phosphorylation site S432 was noted to significantly decrease the generation of radiation-induced single-stranded tails, indicating cell cycle regulation of HR repair by CDK kinase, particularly at the S and G2 phases [41]. Because NHEJ is a dominant pathway in mammalian DSB repair, radiation-sensitive NBS cells could be compromised in NHEJ. Mammalian NHEJ is subdivided into the following two categories: Ku protein-dependent NHEJ (canonical NHEJ) and microhomology-mediated NHEJ (alternative NHEJ). When measured by the end-joining assay using linearized plasmid DNAs, our *Nbs1*-deficient DT40 cells showed normal Ku-dependent NHEJ, suggesting an impairment of the alternative NHEJ, but not the canonical NHEJ. This is consistent with the observation that NBS1 disruption causes MRE11 dysfunction, which impairs the alternative NHEJ [42]. Thus, the MRE11-binding domain of NBS1 at the C-terminus determines cellular radiation sensitivities via HR repair and NHEJ, particularly the alternative NHEJ.

## 4. A Role of NBS1 in Chromatin Remodeling during HR Repair

Chromatin remodeling or the process of decondensing tight chromatin structures is required for many eukaryotic DNA cellular processes such as transcription, replication, and repair. Chromatin remodeling is initiated with several histone modifications, followed by the relocalization of ATP-dependent chromatin remodeling factors, which are classified into the following four families: SWI/SNF, INO80, CHD, and ISWI. Various post-translational histone modifications, such as ubiquitination, acetylation, methylation, and phosphorylation, are known [43]. Among them, a well-characterized modification in DSB repair is the phosphorylation of H2AX, a variant of histone H2A. H2AX is modified by ATM kinase within a few minutes after exposure to IR and is subsequently ubiquitinated by RNF8 and RNF168 so that 53BP1 accumulates at the DSB [35,44,45,46,47,48]. Simultaneously, ATM phosphorylates KAP-1 which, together with phosphorylated H2AX and 53BP1, promotes the dispersion of CHD3.2, a chromatin-remodeling factor that belongs to the CHD family, from heterochromatin regions.

Although similar multiple histone modifications are involved in efficient DSB repair in yeast [49,50,51,52], the experiment using the DSB induction system at the yeast MAT locus revealed an MRX complex-dependent eviction of H2B and H3 from the DSB site [53]. Consistent with this, mammalian NBS1 regulates the release of chromatin-bound H2B from DSB sites when they are generated by the I-PpoI endonuclease [54], suggesting an involvement of NBS1 and histone H2B in chromatin remodeling as a response to DSBs. In agreement, our two-hybrid screening in yeast identified the ubiquitin E3 ligase RNF20 as a novel NBS1-binding partner [27]. RNF20 ubiquitinates the histone H2B, thereby recruiting SNF2h, a chromatin-remodeling factor of the ISWI family. This process is followed by BRCA1 and RAD51 accumulation at DSB sites after IR exposure. Furthermore, this RNF20-dependent chromatin remodeling requires the interaction of RNF20 with the histone chaperon FACT (facilitates chromatin transcription), a heterodimeric complex consisting of SUPT16H (human orthologous of yeast Spt16) and SSRP1 [55,56,57]. Mutation at the interaction of SUPT16H and RNF20 compromised the accumulation of RNF20, SNF2h, BRCA1, and RAD51 at DSB sites. During transcription, FACT is considered to recruit PAF1, a transcription elongation regulator, and RNF20 for the displacement of the H2A-H2B dimer from a nucleosome [55,56,57]. However, the depletion of PAF1 did not affect the accumulation of RNF20 in DSB repair. Consequently, RNF20 initiates the SNF2h-dependent chromatin remodeling during DSB repair by a machinery similar to that of transcription, but independent of PAF1 [58,59]. This model is supported by the observation that when measured by the HR reporter DR-GFP, the frequencies of HR repair were significantly reduced by the depletion of RNF20 or SUPT16H, but not by the depletion of PAF1 [60]. It is noted that the RNF20/SNF2h pathway occurs independently of KAP-1/CHD3.1, which functions along with γH2AX and 53BP1 accumulation at DSB sites. Recently, Klement *et al.* proposed a model in which decondensing heterochromatin at DSB sites required two events; first, CHD3.1 detaches from DSB sites through KAP-1 phosphorylation, and second, SNF2h is replaced with CHD3.1 by RNF20-dependent H2B ubiquitination [61].

## 5. A Role of NBS1 in Initiation of Translesion DNA Synthesis

The first paper that described patients with NBS showed mild photosensitivity [1]. Consistent with this, the cells from NBS patients in Nijmegen showed high sensitivity to UV, although normal UV sensitivity was observed in NBS cells from others [8]. This different sensitivity might be attributed to the different expression level of truncated NBS1 species because the cells showing normal UV sensitivity expressed a high amount of p70 species. Indeed, another paper reported that the depletion of NBS1 by siRNA caused enhanced cellular sensitivity to UV [62], although the mechanism remained to be elucidated. UV induces cyclobutane pyrimidine dimers (CPDs) in genome DNA, causing the collapse or stalling of DNA replication forks during DNA synthesis and eventually leading to cell death [63,64]. These lesions are usually bypassed by translesion DNA synthesis (TLS) polymerases in normal cells, which insert specific nucleotides into a strand that is opposite to the damaged DNA [65]. To date, several TLS polymerases are known in eukaryotes, and these were reported to bypass a variety of lesions with preferences for each inserted nucleotide [66,67]. One of them is Polη TLS polymerase, which is recruited to CPD sites by a polymerase switch with the replication polymerase δ/ε, and it inserts two adenines to the strand opposite the CPD-containing strand [68,69,70]. The polymerase switch is triggered with PCNA mono-ubiquitination by ubiquitin ligase complex RAD6/RAD18 [71,72,73]. We found that UV-induced PCNA mono-ubiquitination is compromised in NBS1-deficient cells so that polymerase switches with Polη, and the resulting TLS were impaired in UV-exposed cells [8]. This was because of the failure of RAD18 recruitment to lesion sites. Indeed, our experiment using NBS1 deletion mutants showed that NBS1 binds to RAD18 at the C-terminus for recruitment to lesion sites. Sequence analysis of amino acids at the C-terminus of NBS1 (650–665 residues) revealed that this RAD18-binding region was well conserved among vertebrates. Moreover, it has several similarities with the RAD18-binding region of the RAD6 protein, suggesting that RAD18 is able to interact with both NBS1 and RAD6 on the same surface. An *in vitro* experiment using recombinant protein confirmed that interaction between NBS1 and RAD18 was remarkably attenuated by the addition of peptides with the same sequence as the RAD18-binding region of RAD6. However, this made us wonder how RAD18 can be recruited by NBS1 and function with RAD6, if binding to NBS1 and RAD6 was mutually exclusive. Interestingly, our co-expression experiments using Myc- and Flag-tagged RAD18 demonstrated the formation of RAD18 homodimers in cells. These homodimers were simultaneously associated with NBS1 and RAD6 during accumulation at lesion sites and function in PCNA ubiquitination. Two correct adenines are preferentially inserted into a strand opposite to the cyclobutane pyrimidine dimers in the Polη-dependent TLS pathway, while Polη belongs to Y-family polymerases with low fidelity. It is known that when Polη is removed, highly frequent mutations are induced after UV exposure and treatment with *N-ethyl-N-*nitrosourea [74]. Similarly, NBS1-deficient cells showed a high frequency of UV-induced mutations [75]; moreover, this mutation spectrum, including transition and transversion, is similar to that of *Polη*-knockout cells, indicating that they function in the same pathway. Thus, NBS1 recruits a RAD18/RAD6 complex to lesion sites by direct binding and initiates the Polη-dependent TLS pathway by PCNA mono-ubiquitination.

## 6. A Role of NBS1 in ICL Repair

Although the cells from patients with NBS have been characterized with high sensitivity to IR and defects in HR repair, some of the patients exhibit high sensitivities to a variety of DNA-damaging agents, including mitomycin-C and cisplatin as well as UV, as described above. Mitomycin-C and cisplatin cause a covalent bond formation between two strands, the so-called ICL DNA damage, which inhibits gene transcription and DNA replication, eventually leading to cell death. This type of lesion is removed by ICL repair machinery, which includes the following: (1) detection of ICL lesions; (2) production of a single-stranded DNA by nicking the DNA downstream and upstream of ICL, resulting in DSB; (3) synthesis of a single-strand DNA opposite the remaining ICLs by TLS; (4) removal of ICL adducts; and (5) rejoining of DSB by HR repair. The genetic disorder of defective ICL repair is known as FA, which is characterized by high sensitivity to ICL agents, such as mitomycin-C, and by predisposition to cancer [76]. FA is divided into at least 17 complement groups from FA-A to FA-T, and all mutated products have been identified, which include HR repair proteins such as BRCA2 (FA-D1), RAD51C (FA-O), and BRCA1 (FA-S). Some patients with NBS display clinical manifestations similar to FA such as aplastic anemia and skeletal anomalies. Both diseases sometimes manifest with microcephaly and increased sensitivity to mitomycin-C [77]; hence, one patient with NBS was misdiagnosed as having FA [78]. Because NBS1 is a HR repair protein, it may be involved in the latter step of ICL repair, namely the rejoining of DSB. However, an unexpected role of NBS1 was shown by our ICL removal assay, which measured the amount of ICL with a dot blot of psoralen-polyethylene oxide-biotin (PPB) cross-linked to DNA [79]. Because HR repair proteins function after the removal of ICLs, the kinetics may be same as those for wild-type cells. The removal kinetics of ICLs in NBS cells was similar to those in cells belonging to FA-A and FA-G, but not in wild-type cells or cells from FA-D1 (BRCA2) [80]. Another possible role of NBS1 in ICL repair is the DNA synthesis of the nicked strand by TLS polymerase, in which NBS1 could recruit Polη to lesion sites, as described in the role of NBS1 in TLS. This explanation is supported by a recent observation that Polη disruption causes high sensitivity to cisplatin because a strand DNA opposite to the remained ICLs is synthesized by Polη [81]. NBS1 may play a role in Polη recruitment to lesion sites during ICL repair, but this needs further confirmatory studies.

Reactive aldehydes, such as acetaldehyde, are common carcinogens that are generated as by-products of several metabolic processes and as constituents/metabolites of food sources such as alcohol [82]. These endogenous mutagens are capable of inducing ICLs [83,84,85]. Langevin *et al.* showed that abnormal development, hematopoietic failure, and cancer predisposition in patients with FA can be attributed to ICLs generated by acetaldehyde metabolism [86]. NBS1-deficient cells are also sensitive to acetaldehydes (Saito *et al.*, unpublished data [87]), and some patients with NBS showed clinical phenotypes such as those of FA [88]. Similar to the FA pathway, NBS1 counteracts endogenous toxic DNA damage; therefore, NBS1 deficiency may be associated with the development of a broad range of malignancies, which are observed in heterozygous carriers of the *NBS1* mutation and some affected persons with NBS.

## 7. Conclusions

**Figure 2:**
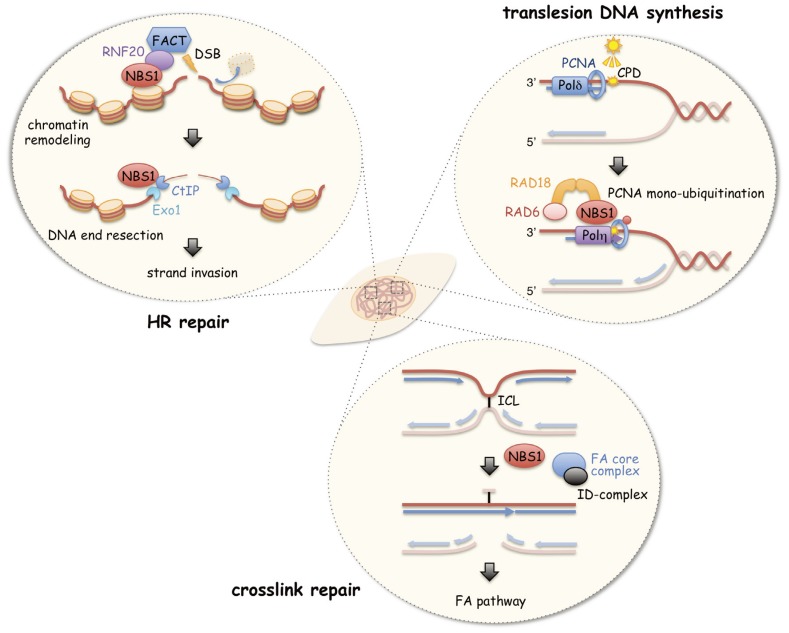
NBS1 acts as a coordinator to maintain the genome integrity. NBS1 cooperates with FACT on RNF20 recruitment and the initiation of HRR. For UV-induced damage, NBS1 interacts with RAD18 and promotes PCNA monoubiquitinaiton for TLS activation. NBS1 also participates in inter-crosslink repair and might counteract the endogenous toxic regions. NBS1 acts as a coordinator responsible for a broad range of DNA damage by orchestrating the following repair proteins to maintain the genome integrity.

NBS1 represents a conserved sequence region within 100 amino acids of the C-terminus where it interacts with ATM, MRE11, RNF20, and RAD18 for several damage responses. Analysis of its binding motifs demonstrated novel functions in addition to its well-established cellular responses such as MRE11-mediated HR repair and ATM-dependent checkpoint regulation. NBS1 regulates chromatin remodeling by RNF20-mediated H2B ubiquitination after IR exposure and results in SNF2h accumulation at DSB sites. After UV exposure, NBS1 binds to RAD18 and initiates Polη-dependent TLS by PCNA ubiquitination. Similarly, NBS1 is involved in ICL repair, possibly by Polη-dependent TLS. Therefore, the depletion of NBS1 causes high sensitivity to IR, sunlight, and endogenous genotoxic agents, including acetaldehyde. Thus, NBS1 seems to coordinate the damage response to protect genome integrity from a vast range of genotoxic agents (Figure 2). NBS1 dysfunction leads to the development of diverse malignancies, as observed in the heterozygous carriers of the *NBS1* mutation, in addition to damage-specific malignancies, such as lymphoma and melanoma.

## References

[1] Weemaes C.M., Hustinx T.W., Scheres J.M., van Munster P.J., Bakkeren J.A., Taalman R.D. (1981). A new chromosomal instability disorder: The Nijmegen breakage syndrome. Acta Paediatr. Scand..

[2] Tauchi H., Matsuura S., Kobayashi J., Sakamoto S., Komatsu K. (2002). Nijmegen breakage syndrome gene, NBS1, and molecular links to factors for genome stability. Oncogene.

[3] Matsuura S., Weemaes C., Smeets D., Takami H., Kondo N., Sakamoto S., Yano N., Nakamura A., Tauchi H., Endo S. (1997). Genetic Mapping Using Microcell-Mediated Chromosome Transfer Suggests a Locus, f.o.r.Nijmegen Breakage Syndrome at Chromosome 8q21–24. Am. J. Hum. Genet..

[4] Matsuura S., Tauchi H., Nakamura A., Kondo N., Sakamoto S., Endo S., Smeets D., Solder B., Belohradsky B.H., der Kaloustian V.M. (1998). Positional cloning of the gene for Nijmegen breakage syndrome. Nat. Genet..

[5] Carney J.P., Maser R.S., Olivares H., Davis E.M., le Beau M., Yates J.R., Hays L., Morgan W.F., Petrini J.H. (1998). The hMre11/hRad50 protein complex and Nijmegen breakage syndrome: Linkage of double-strand break repair to the cellular DNA damage response. Cell.

[6] Varon R., Vissinga C., Platzer M., Cerosaletti K.M., Chrzanowska K.H., Saar K., Beckmann G., Seemanová E., Cooper P.R., Nowak N.J. (1998). Nibrin, a novel DNA double-strand break repair protein, is mutated in Nijmegen breakage syndrome. Cell.

[7] Varon R., Dutrannoy V., Weikert G., Tanzarella C., Antoccia A., Stockl L., Spadoni E., Krüger L.A., di Masi A., Sperling K. (2006). Mild Nijmegen breakage syndrome phenotype due to alternative splicing. Hum. Mol. Genet..

[8] Yanagihara H., Kobayashi J., Tateishi S., Kato A., Matsuura S., Tauchi H., Yamada K., Takezawa J., Sugasawa K., Masutani C. (2011). NBS1 recruits RAD18 via a RAD6-like domain and regulates Pol η-dependent translesion DNA synthesis. Mol. Cell.

[9] Bennett C.B., Lewis A.L., Baldwin K.K., Resnick M.A. (1993). Lethality induced by a single site-specific double-strand break in a dispensable yeast plasmid. Proc. Natl. Acad. Sci. USA.

[10] Wyman C., Kanaar R. (2006). DNA Double-strand break repair: All’s well that ends well. Annu. Rev. Genet..

[11] Symington L.S., Gautier J. (2011). Double-strand break end resection and repair pathway choice. Annu. Rev. Genet..

[12] Sung P., Klein H. (2006). Mechanism of homologous recombination: Mediators and helicases take on regulatory functions. Nat. Rev. Mol. Cell Biol..

[13] Kobayashi J., Iwabuchi K., Miyagawa K., Sonoda E., Suzuki K., Takata M., Tauchi H. (2008). Current topics in DNA double-strand break repair. J. Radiat. Res..

[14] Tauchi H., Kobayashi J., Morishima K., Matsuura S., Nakamura A., Shiraishi T., Ito E., Masnada D., Delia D., Komatsu K. (2001). The forkhead-associated domain of NBS1 is essential for nuclear foci formation after irradiation but not essential for hRAD50-hMRE11-NBS1 complex DNA repair activity. J. Biol. Chem..

[15] Kobayashi J., Tauchi H., Sakamoto S., Nakamura A., Morishima K.-I., Matsuura S., Kobayashi T., Tamai K., Tanimoto K., Komatsu K. (2002). NBS1 localizes to gamma-H2AX foci through interaction with the FHA/BRCT domain. Curr. Biol..

[16] Chapman J.R., Jackson S.P. (2008). Phospho-dependent interactions between NBS1 and MDC1 mediate chromatin retention of the MRN complex at sites of DNA damage. EMBO Rep..

[17] Saito Y., Fujimoto H., Kobayashi J. (2013). Role of NBS1 in DNA damage response and its relationship with cancer development. Transl. Cancer Res..

[18] Shiloh Y., Ziv Y. (2013). The ATM protein kinase: regulating the cellular response to genotoxic stress, and more. Nat. Rev. Mol. Cell Biol..

[19] Williams R.S., Dodson G.E., Limbo O., Yamada Y., Williams J.S., Guenther G., Classen S., Glover J.N., Iwasaki H., Russell P. (2009). Nbs1 Flexibly Tethers Ctp1 and Mre11-Rad50 to Coordinate DNA Double-Strand Break Processing and Repair. Cell.

[20] Maraschio P., Peretti D., Lambiase S., Curto Lo F., Caufin D., Gargantini L., Minoli L., Zuffardi O. (1986). A new chromosome instability disorder. Clin. Genet..

[21] Tauchi H., Kobayashi J., Morishima K.-I., van Gent D.C., Shiraishi T., Verkaik N.S., van Heems D., Ito E., Nakamura A., Sonoda E. (2002). Nbs1 is essential for DNA repair by homologous recombination in higher vertebrate cells. Nature.

[22] Sakamoto S., Iijima K., Mochizuki D., Nakamura K., Teshigawara K., Kobayashi J., Matsuura S., Tauchi H., Komatsu K. (2007). Homologous recombination repair is regulated by domains at the N- and C-terminus of NBS1 and is dissociated with ATM functions. Oncogene.

[23] Ohta K., Nicolas A., Furuse M., Nabetani A., Ogawa H., Shibata T. (1998). Mutations in the MRE11, RAD50, XRS2, and MRE2 genes alter chromatin configuration at meiotic DNA double-stranded break sites in premeiotic and meiotic cells. Proc. Natl. Acad. Sci. USA.

[24] Haber J.E. (1998). The many interfaces of Mre11. Cell.

[25] Furuse M., Nagase Y., Tsubouchi H., Murakami-Murofushi K., Shibata T., Ohta K. (1998). Distinct roles of two separable *in vitro* activities of yeast Mre11 in mitotic and meiotic recombination. EMBO J..

[26] Falck J., Coates J., Jackson S.P. (2005). Conserved modes of recruitment of ATM, ATR and DNA-PKcs to sites of DNA damage. Nature.

[27] Nakamura K., Kato A., Kobayashi J., Yanagihara H., Sakamoto S., Oliveira D.V., Shimada M., Tauchi H., Suzuki H., Tashiro S. (2011). Regulation of homologous recombination by RNF20-dependent H2B ubiquitination. Mol. Cell.

[28] Oliveira D.V., Kato A., Nakamura K., Ikura T., Okada M., Kobayashi J., Yanagihara H., Saito Y., Tauchi H., Komatsu K. (2014). Histone chaperone FACT regulates homologous recombination by chromatin remodeling through interaction with RNF20. J. Cell Sci..

[29] Demuth I. (2004). An inducible null mutant murine model of Nijmegen breakage syndrome proves the essential function of NBS1 in chromosomal stability and cell viability. Hum. Mol. Genet..

[30] Yamaguchi-Iwai Y., Sonoda E., Sasaki M.S., Morrison C., Haraguchi T., Hiraoka Y., Yamashita Y.M., Yagi T., Takata M., Price C. (1999). Mre11 is essential for the maintenance of chromosomal DNA in vertebrate cells. EMBO J..

[31] Zhu J., Petersen S., Tessarollo L., Nussenzweig A. (2001). Targeted disruption of the Nijmegen breakage syndrome gene NBS1 leads to early embryonic lethality in mice. Curr. Biol..

[32] Buerstedde J.M., Takeda S. (1991). Increased ratio of targeted to random integration after transfection of chicken B cell lines. Cell.

[33] Johnson R.D., Liu N., Jasin M. (1999). Mammalian XRCC2 promotes the repair of DNA double-strand breaks by homologous recombination. Nature.

[34] Celeste A., Petersen S., Romanienko P.J., Fernandez-Capetillo O., Chen H.-T., Sedelnikova O.A., Reina-San-Martin B., Coppola V., Meffre E., Difilippantonio M.J. (2002). Genomic instability in mice lacking histone H2AX. Science.

[35] Bassing C.H., Chua K.F., Sekiguchi J., Suh H., Whitlow S.R., Fleming J.C., Monroe B.C., Ciccone D.N., Yan C., Vlasakova K. (2002). Increased ionizing radiation sensitivity and genomic instability in the absence of histone H2AX. Proc. Natl. Acad. Sci. USA.

[36] White C.I., Haber J.E. (1990). Intermediates of recombination during mating type switching in Saccharomyces cerevisiae. EMBO J..

[37] Sartori A.A., Lukas C., Coates J., Mistrik M., Fu S., Bartek J., Baer R., Lukas J., Jackson S.P. (2007). Human CtIP promotes DNA end resection. Nature.

[38] Garcia V., Phelps S.E.L., Gray S., Neale M.J. (2011). Bidirectional resection of DNA double-strand breaks by Mre11 and Exo1. Nature.

[39] Shibata A., Moiani D., Arvai A.S., Perry J., Harding S.M., Genois M.-M., Maity R., van Rossum-Fikkert S., Kertokalio A., Romoli F. (2014). DNA Double-Strand Break Repair Pathway Choice Is Directed by Distinct MRE11 Nuclease Activities. Mol. Cell.

[40] Dodson G.E., Limbo O., Nieto D., Russell P. (2010). Phosphorylation-regulated binding of Ctp1 to Nbs1 is critical for repair of DNA double-strand breaks. Cell Cycle.

[41] Falck J., Forment J.V., Coates J., Mistrik M., Lukas J., Bartek J., Jackson S.P. (2012). CDK targeting of NBS1 promotes DNA-end resection, replication restart and homologous recombination. EMBO Rep..

[42] Xie A., Kwok A., Scully R. (2009). Role of mammalian Mre11 in classical and alternative nonhomologous end joining. Nat. Struct. Mol. Biol..

[43] Van Attikum H., Gasser S.M. (2005). The histone code at DNA breaks: A guide to repair?. Nat. Rev. Mol. Cell Biol..

[44] Stewart G.S., Wang B., Bignell C.R., Taylor A.M.R., Elledge S.J. (2003). MDC1 is a mediator of the mammalian DNA damage checkpoint. Nature.

[45] Mailand N., Bekker-Jensen S., Faustrup H., Melander F., Bartek J., Lukas C., Lukas J. (2007). RNF8 ubiquitylates histones at DNA double-strand breaks and promotes assembly of repair proteins. Cell.

[46] Kolas N.K., Chapman J.R., Nakada S., Ylanko J., Chahwan R., Sweeney F.D., Panier S., Mendez M., Wildenhain J., Thomson T.M. (2007). Orchestration of the DNA-damage response by the RNF8 ubiquitin ligase. Science.

[47] Doil C., Mailand N., Bekker-Jensen S., Menard P., Larsen D.H., Pepperkok R., Ellenberg J., Panier S., Durocher D., Bartek J. (2009). RNF168 binds and amplifies ubiquitin conjugates on damaged chromosomes to allow accumulation of repair proteins. Cell.

[48] Daley J.M., Sung P. (2014). 53BP1, BRCA1 and the choice between recombination and end joining at DNA double-strand breaks. Mol. Cell. Biol..

[49] Ikura T., Ogryzko V.V., Grigoriev M., Groisman R., Wang J., Horikoshi M., Scully R., Qin J., Nakatani Y. (2000). Involvement of the TIP60 histone acetylase complex in DNA repair and apoptosis. Cell.

[50] Van Attikum H., Fritsch O., Hohn B., Gasser S.M. (2004). Recruitment of the INO80 complex by H2A phosphorylation links ATP-dependent chromatin remodeling with DNA double-strand break repair. Cell.

[51] Murr R., Loizou J.I., Yang Y.-G., Cuenin C., Li H., Wang Z.-Q., Herceg Z. (2005). Histone acetylation by Trrap-Tip60 modulates loading of repair proteins and repair of DNA double-strand breaks. Nat. Cell Biol..

[52] Niida H., Katsuno Y., Sengoku M., Shimada M., Yukawa M., Ikura M., Ikura T., Kohno K., Shima H., Suzuki H. (2010). Essential role of Tip60-dependent recruitment of ribonucleotide reductase at DNA damage sites in DNA repair during G1 phase. Genes Dev..

[53] Tsukuda T., Fleming A.B., Nickoloff J.A., Osley M.A. (2005). Chromatin remodelling at a DNA double-strand break site in Saccharomyces cerevisiae. Nature.

[54] Berkovich E., Monnat R.J., Kastan M.B. (2007). Roles of ATM and NBS1 in chromatin structure modulation and DNA double-strand break repair. Nat. Cell Biol..

[55] Orphanides G., Wu W.H., Lane W.S., Hampsey M., Reinberg D. (1999). The chromatin-specific transcription elongation factor FACT comprises human SPT16 and SSRP1 proteins. Nature.

[56] Winkler D.D., Luger K. (2011). The histone chaperone FACT: Structural insights and mechanisms for nucleosome reorganization. J. Biol. Chem..

[57] Soria G., Polo S.E., Almouzni G. (2012). Prime, repair, restore: The active role of chromatin in the DNA damage response. Mol. Cell.

[58] Belotserkovskaya R., Oh S., Bondarenko V.A., Orphanides G., Studitsky V.M., Reinberg D. (2003). FACT facilitates transcription-dependent nucleosome alteration. Science.

[59] Heo K., Kim H., Choi S.H., Choi J., Kim K., Gu J., Lieber M.R., Yang A.S., An W. (2008). FACT-mediated exchange of histone variant H2AX regulated by phosphorylation of H2AX and ADP-ribosylation of Spt16. Mol. Cell.

[60] Piro A.S., Mayekar M.K., Warner M.H., Davis C.P., Arndt K.M. (2012). Small region of Rtf1 protein can substitute for complete Paf1 complex in facilitating global histone H2B ubiquitylation in yeast. Proc. Natl. Acad. Sci..

[61] Klement K., Luijsterburg M.S., Pinder J.B., Cena C.S., del Nero V., Wintersinger C.M., Dellaire G., van Attikum H., Goodarzi A.A. (2014). Opposing ISWI- and CHD-class chromatin remodeling activities orchestrate heterochromatic DNA repair. J. Cell Biol..

[62] Biard D.S.F. (2007). Untangling the relationships between DNA repair pathways by silencing more than 20 DNA repair genes in human stable clones. Nucleic Acids Res..

[63] Cordeiro-Stone M., Zaritskaya L.S., Price L.K., Kaufmann W.K. (1997). Replication fork bypass of a pyrimidine dimer blocking leading strand DNA synthesis. J. Biol. Chem..

[64] Ling H., Boudsocq F., Plosky B.S., Woodgate R., Yang W. (2003). Replication of a cis-syn thymine dimer at atomic resolution. Nature.

[65] Lehmann A.R. (2006). New functions for Y family polymerases. Mol. Cell.

[66] Masutani C., Araki M., Yamada A., Kusumoto R., Nogimori T., Maekawa T., Iwai S., Hanaoka F. (1999). Xeroderma pigmentosum variant (XP-V) correcting protein from HeLa cells has a thymine dimer bypass DNA polymerase activity. EMBO J..

[67] Masutani C., Kusumoto R., Yamada A., Dohmae N., Yokoi M., Yuasa M., Araki M., Iwai S., Takio K., Hanaoka F. (1999). The XPV (xeroderma pigmentosum variant) gene encodes human DNA polymerase eta. Nature.

[68] Masutani C., Kusumoto R., Iwai S., Hanaoka F. (2000). Mechanisms of accurate translesion synthesis by human DNA polymerase eta. EMBO J..

[69] McCulloch S.D., Kokoska R.J., Masutani C., Iwai S., Hanaoka F., Kunkel T.A. (2004). Preferential cis-syn thymine dimer bypass by DNA polymerase eta occurs with biased fidelity. Nature.

[70] Yang X.H., Zou L. (2009). Dual functions of DNA replication forks in checkpoint signaling and PCNA ubiquitination. Cell Cycle.

[71] Watanabe K., Tateishi S., Kawasuji M., Tsurimoto T., Inoue H., Yamaizumi M. (2004). Rad18 guides poleta to replication stalling sites through physical interaction and PCNA monoubiquitination. EMBO J..

[72] Tissier A., Kannouche P., Reck M.-P., Lehmann A.R., Fuchs R.P.P., Cordonnier A. (2004). Co-localization in replication foci and interaction of human Y-family members, DNA polymerase pol eta and REVl protein. DNA Repair.

[73] Karras G.I., Jentsch S. (2010). The RAD6 DNA damage tolerance pathway operates uncoupled from the replication fork and is functional beyond S Phase. Cell.

[74] Busuttil R.A., Lin Q., Stambrook R.J., Kucherlapati R., Vijg J. (2008). Mutation frequencies and spectra in DNA polymerase η-deficient mice. Cancer Res..

[75] Gondo Y., Shioyama Y., Nakao K., Katsuki M. (1996). A novel positive detection system of *in vivo* mutations in rpsL (strA) transgenic mice. Mutat. Res..

[76] Moldovan G.-L., D’Andrea A.D. (2009). How the Fanconi anemia pathway guards the genome. Annu. Rev. Genet..

[77] Nakanishi K., Taniguchi T., Ranganathan V., New H.V., Moreau L.A., Stotsky M., Mathew C.G., Kastan M.B., Weaver D.T., D’Andrea A.D. (2002). Interaction of FANCD2 and NBS1 in the DNA damage response. Nat. Cell Biol..

[78] Gennery A.R., Slatter M.A., Bhattacharya A., Barge D., Haigh S., O’Driscoll M., Coleman R., Abinun M., Flood T.J., Cant A.J. (2004). The clinical and biological overlap between Nijmegen Breakage Syndrome and Fanconi anemia. Clin. Immunol..

[79] Zhang Y., Price B.D., Tetradis S., Chakrabarti S., Maulik G., Makrigiorgos G.M. (2001). Reproducible and inexpensive probe preparation for oligonucleotide arrays. Nucleic Acids Res..

[80] Tsuchida K., Komatsu K. (2008). Impaired removal of DNA interstrand cross-link in Nijmegen breakage syndrome and Fanconi anemia, but not in BRCA-defective group. Cancer Sci..

[81] Lee Y.S., Gregory M.T., Yang W. (2014). Human Polz purified with accessory subunits is active in translesion DNA synthesis and complements Polh in cisplatin bypass. Proc. Natl. Acad. Sci. USA.

[82] O’Brien P.J., Siraki A.G., Shangari N. (2005). Aldehyde sources, metabolism, molecular toxicity mechanisms, and possible effects on human health. Crit. Rev. Toxicol..

[83] Wang M., McIntee E.J., Cheng G., Shi Y., Villalta P.W., Hecht S.S. (2000). Identification of DNA adducts of acetaldehyde. Chem. Res. Toxicol..

[84] Seitz H.K., Becker P. Alcohol metabolism and cancer risk. http://pubs.niaaa.nih.gov/publications/arh301/38-47.pdf.

[85] Nagayoshi H., Matsumoto A., Nishi R., Kawamoto T., Ichiba M., Matsuda T. (2009). Increased formation of gastric N2-ethylidene-2'-deoxyguanosine DNA adducts in aldehyde dehydrogenase-2 knockout mice treated with ethanol. Mutat. Res. Genet. Toxicol. Environ. Mutagen..

[86] Langevin F., Crossan G.P., Rosado I.V., Arends M.J., Patel K.J. (2011). Fancd2 counteracts the toxic effects of naturally produced aldehydes in mice. Nature.

[87] Saito Y., Kobayashi J., Komatsu K. (2015).

[88] Chrzanowska K.H., Gregorek H., Dembowska-Bagińska B., Kalina M.A., Digweed M. (2012). Nijmegen breakage syndrome (NBS). Orphanet J. Rare Dis..

